# A feasibility trial of pulmonary rehabilitation for patients with COPD in a low resource setting: Jaffna, Sri Lanka

**DOI:** 10.1186/s12890-022-02092-x

**Published:** 2022-08-08

**Authors:** Mathanki Sooriyakanthan, Mark W. Orme, Kanagasabai Sivapalan, Gowry Selvaratnam, Sally J. Singh, Savithri Wimalasekera

**Affiliations:** 1 Department of Physiology, Faculty of Medicine, University of Jaffna, Jaffna, Sri Lanka; 2 Department of Respiratory Sciences, University of Leicester, Leicester, UK; 3 Centre for Exercise and Rehabilitation Science, NIHR Leicester Biomedical Research Centre-Respiratory, University Hospitals of Leicester NHS Trust, Leicester, UK; 4 Department of Medicine, Faculty of Medicine, University of Jaffna, Jaffna, Sri Lanka; 5 Department of Physiology, Faculty of Medical Sciences, University of Sri Jayewardenepura, Jaffna, Sri Lanka

**Keywords:** COPD, Pulmonary rehabilitation, Feasibility, Education, Exercise, Low- and middle-income countries

## Abstract

**Background:**

Pulmonary rehabilitation is recommended for most patients with chronic obstructive pulmonary disease (COPD). Accordingly, the aim of this study was to explore the feasibility of devising a pulmonary rehabilitation program for patients with COPD in a low resource setting (Jaffna, Sri Lanka) and to observe its effects.

**Methods:**

Non-randomized controlled feasibility trial of ambulatory patients with COPD attending the pulmonary outpatient clinic of the Jaffna Teaching Hospital, Northern Province, Sri Lanka. Age-matched patients were allocated alternatively to an intervention group or to a control group. Twice weekly, for six weeks, patients in the intervention group attended pulmonary rehabilitation sessions consisting of supervised stretching, aerobic and strengthening exercises, and patient-education. Before and at the conclusion of the study, all patients performed incremental shuttle walking test (ISWT), 6-min walk test (6MWT) and completed the Medical Research Council (MRC) dyspnea scale, COPD assessment test (CAT), chronic COPD questionnaire (CCQ), and hospital anxiety depression scale (HADS).

**Results:**

204 patients with COPD (94% males) were identified at screening; 136 (66.7%) were eligible for pulmonary rehabilitation and 96 patients (47%) consented to participate. Of these, 54 patients (53 males) eventually participated in the study (42 patients were discouraged to participate by family members or friends); 40 patients (20 in the rehabilitation group and 20 patients in the control group) completed the study. Baseline characteristics of the intervention group and the control group were similar. 95% of patients in the intervention group adhered to regular home training exercises (self-reported diary). At post assessment, only the intervention group experienced clinically-meaningful improvements in symptoms and exercise capacity.

**Conclusion:**

A simple and clinically beneficial pulmonary rehabilitation program for patients with COPD can be effectively implemented in a low resource setting. However, there is a need for educating patients and the local community on the benefits of pulmonary rehabilitation to enhance uptake.

Retrospective Trial Registration date and number: 16/04/2021, ISRCTN10069208.

## Introduction

Chronic Obstructive Pulmonary Disease (COPD) is a major cause of morbidity and mortality worldwide, contributing to 6% of global deaths [1]. It is estimated that the burden of COPD will continue to rise due to increasing exposure to risk factors, with most of the burden in low- and middle-income countries (LMIC). The prevalence of COPD in Sri Lanka is estimated at 10.5% [2]. Unfortunately, due to lack of access to spirometry testing and the absence of community screening programs COPD is largely underdiagnosed in the country.

Dyspnea, cough and sputum production, common symptoms of COPD, reduce health-related quality of life (HRQoL) and ability to engage in employment [1, 3]. COPD is also associated with co-morbidities that can lead to extra-pulmonary manifestations of the disease including weight loss, and skeletal muscle dysfunction [4]. To avoid unpleasant symptoms, patients with COPD often refrain from physical activity, which ultimately leads to further muscle deconditioning and worse dyspnea. This triggers a vicious cycle unless physical activities are maintained []. The individual and societal impact of COPD demands interventions to improve HRQoL such as pulmonary rehabilitation [1, 6, 7].

In high-income countries is has been reported that pulmonary rehabilitation has both clinical and health-economic benefits [6]. In LMIC, such as Sri Lanka, resources for- and awareness of pulmonary rehabilitation are limited [8]. Not surprisingly, despite growing interest [9, 10], there have been no studies of pulmonary rehabilitation in Sri Lanka, a country in which patients with COPD are managed, at most, with pharmacotherapy alone.

The primary aim of our study was thus to test the feasibility of conducting pulmonary rehabilitation in a low resource unit in a Sri Lanka teaching hospital. The secondary aim of the study was to describe the effect of pulmonary rehabilitation on respiratory function, symptoms scores, exercise capacity, and psychological wellbeing for patients with COPD.

## Methodology

### Study design and registration

This non-randomized controlled trial was carried out at the Jaffna Teaching Hospital in Sri Lanka between June 2019 and March 2020. The Ethical Review Committee of the Faculty of Medical Sciences, University of Sri Jayewardenepura (Ref.No:18/35) approved the study and written informed consent was obtained from all participants. The study was retrospectively registered at the International Standard Randomized Controlled Trial Number (16/04/2021, ISRCTN10069208).

### Site set-up

**Fig. 1:**
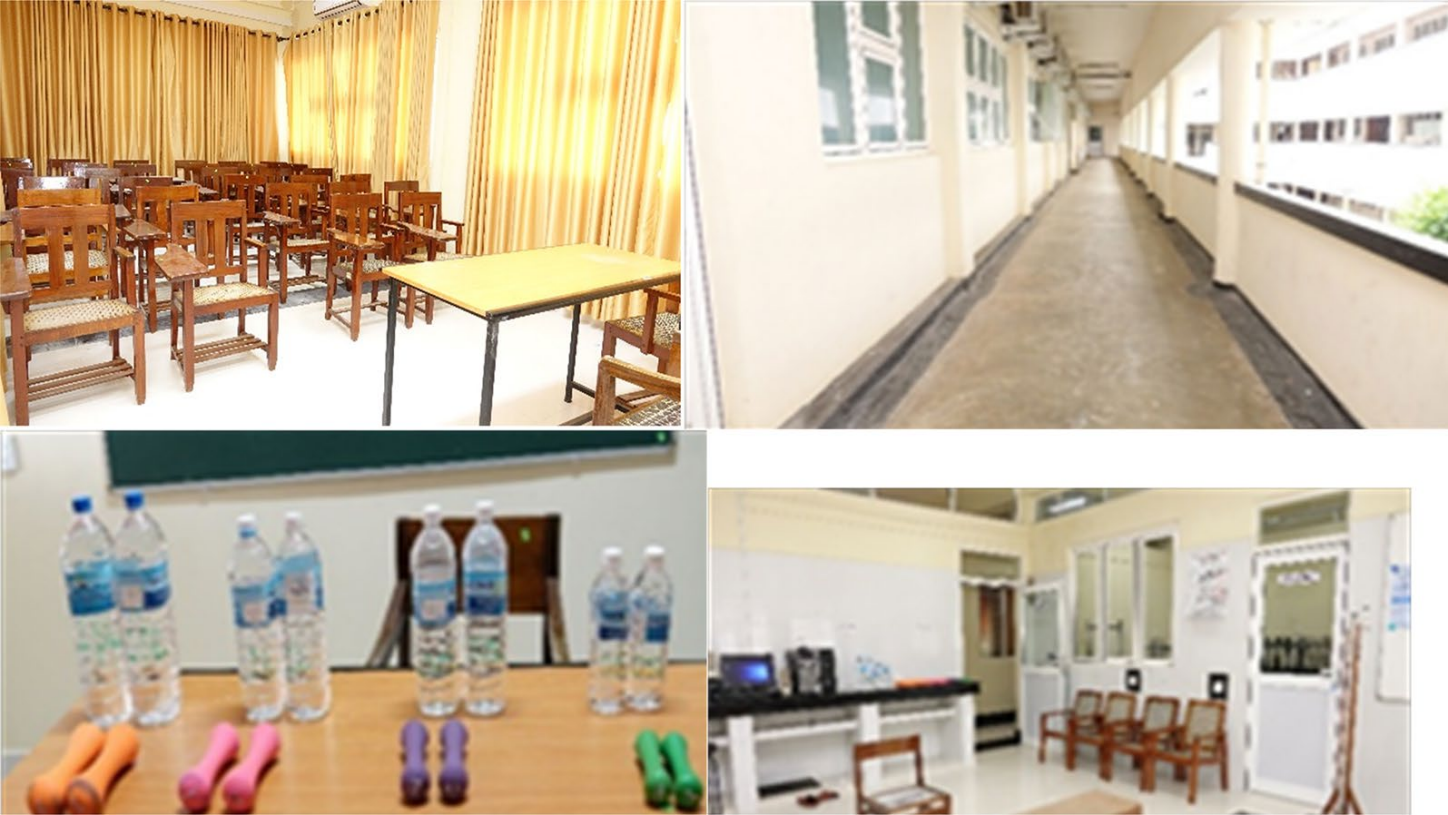
Sites for pulmonary rehabilitation. *(Left upper panel)* Tutorial room. *(Right upper panel)* Outdoor corridor for aerobic/walking exercise training. *(Left lower panel)* Four stations for strength training. *(Right lower panel)* Chairs for patients to rest

As there was no established pulmonary rehabilitation service, the minimum necessary equipment was purchased. Arrangement for referral in case of medical emergencies occurring during pulmonary rehabilitation sessions was established with the chest physician of the University of Jaffna. A tutorial room was repurposed for pulmonary rehabilitation education sessions (Fig. 1). The adjacent outdoor corridor was used as the space for aerobic/walking exercise training. Strength training exercises were conducted in the laboratory of the Department of Physiology. Four stations for strength training exercises were equipped with three pairs of dumbbells spanning 500 g, 750 g, 1 kg, and 1.5 kg. One chair was available for the sit-to-stand exercise station. A wooden step (15 cm height) was assembled for the step-ups station. Another two chairs were placed to mark the stations for biceps curls and pull-ups. Rehabilitation sessions were attended by 2–6 patients at a time. Chairs were also available for patients to rest.

### Participants

#### Inclusion criteria

Moderate-to-severe COPD (Table 2); ⩾18 years of age; Medical Research Council (MRC) dyspnea scale ≥ 2; exercise intolerance; stable clinical condition.

#### Exclusion criteria

Severe or unstable cardiovascular diseases. (Patients with stable angina were not excluded from the study). Additional exclusion criteria were: lung diseases other than COPD, peripheral neuropathies, stroke, musculoskeletal dysfunction, hearing impairment and psychological conditions limiting participation.

### Recruitment procedure

Patients were contacted while attending their routine medical clinics at the Jaffna Teaching Hospital. Patients who met study criteria were invited to participate. Patients wishing to take part in the study attended a baseline assessment where they gave written informed consent.

To recruit as many patients as possible, investigators explained to the patients the potential benefits of pulmonary rehabilitation. Most patients were unemployed. Accordingly, at the time of screening, patients were informed that they would be reimbursed transportation costs for each study visit.

### Usual care

All study participants were advised to adhere to usual medical management that could include inhaled long-acting beta agonist or long-acting anti muscarinic or inhaled corticosteroids plus as-needed inhaled short-acting beta agonists (e.g., salbutamol).

### Pulmonary rehabilitation (Intervention group only)

The pulmonary rehabilitation protocol was based on the protocol of the University Hospitals of Leicester, UK modified for a low resource setting [11]. Before study commencement, the principal investigator (MS) met with a team from Leicester University (MWO and SJS) who visited Sri Lanka to conduct face-to-face training workshops. All rehabilitation sessions were supervised by the principal investigator (MS) who was assisted by other two staff member of the Department of Physiology.

Supervised pulmonary rehabilitation sessions were conducted twice weekly for 6 weeks. Each session lasted for about 2 h (1 h of supervised exercise and 1 h of group education). Stretching exercises were performed initially, followed by supervised walking. Patients were instructed to start walking slowly first and then to speed up and again to slow aiming for a Borg dyspnea score of 3–6 (moderate to severe dyspnea) [12, 13]. Patients were instructed to stop walking whenever they experienced symptoms such as dizziness, feeling faint, blurred vision, or severe breathlessness. Walking time was increased gradually at each session with breaks for those who could not walk 30 min continuously during the first day. Patients were instructed to complete daily training walks at home.

After walking, patients performed strength training exercises which consisted of biceps curls, pull-ups, sit-to-stand and step-up exercises. Each exercise was carried out for three sets of 10 repetitions. Biceps curls were carried out using dumbbells weighing 500 g, 750 g, 1 kg and 1.5 kg. Biceps curls were started using 500 g weight initially. If the patient was able to achieve 30 repetitions within 2 min, the weight was increased in the next session. In addition to the twice weekly supervised sessions, patients were instructed to perform strength training exercises also twice weekly at home. To exercise at home, patients were given 2 bottles filled with enough water to equal the weight of the dumbbells used in the exercise laboratory. Compliance to the home exercise regimen was checked at each subsequent pulmonary rehabilitation session based on self-report, recorded as a diary entry by the principal investigator.

During each visit to the Department of Physiology, patients in the intervention group participated in education sessions covering the following topics: disease education, benefits of exercise, dietary advice, relaxation, energy conservation, avoidance of exacerbations, medication, chest clearance and managing breathlessness. This educational material was based on the one developed by the University Hospitals of Leicester, UK. Printed handouts were also given to all participants at the end of each education session. Although most participants were illiterate, we thought it worth providing this material hoping that a family member or a caregivers might have been able to read the material to the patients.

### Outcomes

To assess the feasibility of conducting pulmonary rehabilitation in a low resource unit (primary outcome) we recorded the parameters listed in Table 1. To describe the effect of our pulmonary rehabilitation program on respiratory function, symptoms scores, exercise capacity, and psychological wellbeing in patients with COPD (secondary outcome) before and at the conclusion of the study, all patients underwent incremental shuttle walking test (ISWT) and 6-min walk test (6MWT). In all patients we also recorded MRC dyspnea scale, COPD assessment test (CAT), chronic COPD questionnaire (CCQ), and hospital anxiety depression scale (HADS).

**Table 1:** Measures of feasibility

Feasibility measures	Criteria
Suitability of inclusion criteria	Proportion of ineligible patients, reasons for ineligibility
Refusal to participate	Proportion of eligible patients not consenting to participate, reasons for declining
Uptake and completion of the study	Proportion of patients enrolled into the study and number of patients who completed the 6-weeks program, reasons for not completing the program
Compliance to pulmonary rehabilitation sessions	Proportion of the 12 scheduled classes attended
Adherence to home exercise	Self-report exercise diary assessed via a self-report exercise diary

### Group allocation

After the baseline visit, sex, age-matched COPD patients were alternatively allocated to intervention and control groups. Patients were stratified according to gender and age groups (41–50, 51–60, 61–70, 70–80). Due to limited staffing, it was not possible to blind the investigators to group allocation for end-of-study measures. In addition, it was not possible to blind participants to the intervention due to the nature of pulmonary rehabilitation.

### Secondary outcomes

#### Respiratory symptom burden

The MRC dyspnoea scale [14] with mean Minimum Clinically Important Difference (MCID) of 1 [15], CAT [16] with mean MCID of 2 [170] and CCQ [181] with mean MCID of 0.2 [19] were translated and used to record symptoms scores. Although these questionnaires were designed to be self-administered, this was not possible in our population due to low literacy levels. Therefore, the questions and responses were read to the patients by a single observer and the patient responses were marked.

#### Exercise capacity

Exercise capacity was assessed by means of 6-MWT [20] and ISWT [21). Two tests were performed to assess exercise capacity by ISWT. Peripheral oxygen saturation (SpO_2_), blood pressure, heart rate, Borg dyspnea scale [13] and Borg exertion scale [13] were recorded at the beginning and end of each test. The distance walked was measured at the end of test, with the greatest distance of the two ISWT tests carried forward.

#### Psychological wellbeing (anxiety and depression)

The HADS questionnaire [22] was translated and used as an interviewer-administered questionnaire. Questions and potential responses were read to each patient and the patient’s response for each question was marked. The MCID of the HADS is 2 [23].

## Statistical analysis

Categorical variables were reported as percentages and continuous variables as mean ± SD or as medians and interquartile ranges (IQRs). Comparison of continuous variables was performed using paired T test and Wilcoxon signed-rank test when comparing two dependent samples (intervention or controls). Statistical significance was assumed at two-tailed *p* values of less than 0.05. This is a feasibility study. Accordingly, no formal sample size calculation was performed. We aimed to recruit 50–60 COPD patients (25–30 per group). All analyses were done using SPSS 20 (IBM, Chicago, USA).

## Results

### Trial feasibility

**Fig. 2:**
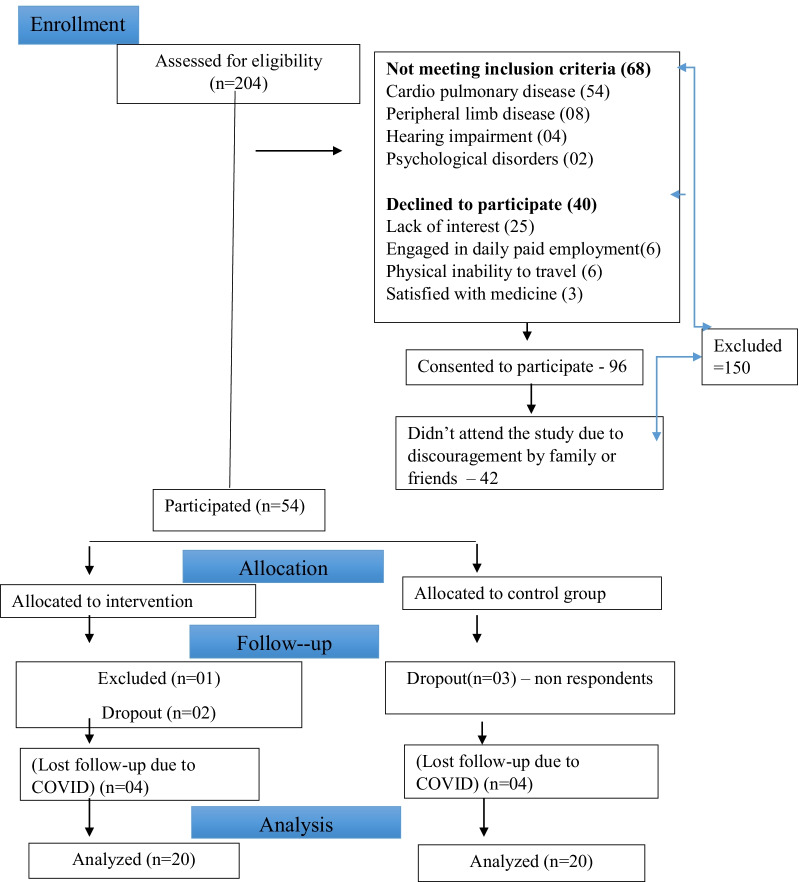
Flowchart illustrating recruitment of study participants

A total of 204 patients (94% male) were identified during the study period (Fig. 2). Out of 136 (66.7%) of them were eligible for the study. Main reasons for exclusions were: cardio pulmonary diseases (mainly unstable angina) (54 out of 204 or 26.5%), limb disorders or rheumatoid arthritis affecting walking (8 out of 204 or 3.9%), hearing impairment (4 out of 204 or 2.0%), and psychological disorders (2 out of 204 or 1.0%). Among eligible patients, 96 (70.6%) consented to participate at the time of screening. Reasons for refusal were lack of interest (18.3%), engagement in daily paid employment (4.4%), physical inability to travel or need of by-standers to accompany (4.4%) and believing pulmonary rehabilitation was unnecessary (2.2%) (Fig. 2).

Investigators tried their best to accommodate patients’ requests for date and time of baseline assessment yet only 54 patients (53 males and 1 female) of the 96 who had consented to the study attended the baseline assessment visit. The remaining 42 patients did not come back for baseline assessment. When contacted, these patients stated that they had been discouraged to do so by family members or friends.

The study was interrupted at the outbreak of the COVID-19 pandemic, after recruiting 54 patients (27 in the intervention group and 27 in the control group, Fig. 2). Out of 27 patients in the intervention group, one was excluded due to inability to cope with walking tests and two dropped out (7.4%) from the study. There were three dropouts in the control group. Eight patients were lost at follow up due to the outbreak of the COVID-19 pandemic. As a result, data of 40 patients (20 in the intervention group and 20 in the control group and all male) who completed the study were analyzed (Fig. 2).

All participants were compliant to the supervised pulmonary rehabilitation sessions. Two patients residing far from the Jaffna Teaching Hospital missed rehabilitation sessions due to the rainy season. Those two patients had the pulmonary rehabilitation sessions extended to 8 weeks until they completed 12 supervised sessions. No patient experienced complications associated to pulmonary rehabilitation. 95% of patients in the intervention group claimed adherence to regular home training exercise (self-reported diary).

### Participant characteristics

As summarized in Table 2, baseline characteristics such as age, height, weight, BMI, smoking status and number of co-morbidities of the intervention and control groups were broadly comparable (Table 2).

**Table 2:** Baseline patient characteristics

Baseline characteristics	Intervention group (n = 20)	Control group (n = 20)
Age (years)	67.1 ± 6.8	68.5 ± 6.5
Height (cm)	162.9 ± 9.0	163.3 ± 4.6
Weight (kg)	55.3 ± 15.6	53.1 ± 8.3
BMI (kg/m^2^)	20.78 ± 5.25	19.96 ± 3.08
Smoking (pack years)	12.5 (2.1–20.0)^a^	15.0 (2.5–3.5)^a^
Comorbidities (number)	1.0 (0.0–1.8)^a^	1. 0 (0.0–1.0)^a^
FVC (L)	1.9 ± 0.4	2. 1 ± 0.5
FEV_1_ (L)	1.1 ± 0.3	1. 2 ± 0.3
FVC (% predicted)	69.6 ± 13.6	77.9 ± 14.5
FEV_1_ (% predicted)	47.9 ± 12.5	51.4 ± 12.2
FEV_1_/FVC	62.2 (48.1- 67.2)^a^	58.75 (49.6- 63.1)^a^
PEFR (L/min)	193.0 ± 66.0	218.0 ± 60.3
PEF (% predicted)	46.9 ± 13.6	53.7 ± 15.5
MRC	3. 3 ± 1.1	2.9 ± 0.9
CAT	18.6 ± 7.9	18.2 ± 7.6
CCQ	2.5 ± 0.9	2.4 ± 1.0
HADS-Anxiety	7.6 ± 3.8	6.6 ± 4.0
HADS-Depression	4.0 (3.0–7.78)^a^	7.0 (4.0–11.8)^a^
6MWD (m)	409.8 ± 6.2	396.7 ± 94.2
ISWD (m)	223.0 ± 63.1	216.0 ± 88.2

*FVC* forced vital capacity, *FEV*_1_ forced expiratory volume in the first second, *PEF* peak expiratory flow, *MRC* Medical Research Council, *CAT* COPD assessment test, *CCQ*-Chronic COPD questionnaire, *HADS* hospital anxiety and depression scale, *6MWD* six minute walk distance, *ISWD* incremental shuttle walk distance

^a^Median (IQR)

### Changes at study completion

Symptoms scores assessed by CCQ and CAT showed an improvement at least four times the MCID [17, 19] (Table 3). The mean improvement in MRC dyspnea scale also was greater than MCID [15]. Improvement in HADS-anxiety score crossed the MCID, but not the reduction in HADS depression [23] (Table 3). Exercise performance following pulmonary rehabilitation program was closer to- or better than- MCIDs for 6MWD and ISWD [24, 25] (Table 4). Nineteen (95%), 18 (90%), 10 (50%) and 9 (45%) out of 20 patients in the intervention group experienced improvements in CAT, CCQ, ISWT and 6MWT at or beyond the respective MCIDs. No patient in the control group experienced MCID improvements in 6MWD, ISWD, MRC, CAT, CCQ, HADS-anxiety and HADS-depression (Tables 3 and 4).

**Table 3:** Respiratory symptoms and psychological wellbeing at baseline and at the completion of the study

Scores	Intervention group (n = 20)		Control Group (n = 20)	
	Baseline	Follow-Up	Baseline	Follow-Up
MRC	3. 3 ± 1.1	1.8 ± 0.6*	2.9 ± 1.0	3.0 ± 1.1
CAT	18.6 ± 7.9	7.5 ± 3.7*	18.1 ± 7.6	18.4 ± 7.6
CCQ (total)	2.6 (1.8–3.0)	0.8(0.6–1.4)	2.3 ± 0.9	2.2 ± 1.0
CCQ (symptom)	2.6 (2.2–3.0)	1.3 ± 0.8*	2.4 ± 1.2	2.4 ± 1.2
CCQ (functional)	2.7 (2.2–3.0)	0. 8(0.6–0.7)*	2.6 ± 1.3	2.6 ± 1.5
CCQ (mental score)	1.5(0.0–2.8)	0. 0(0.0- 0.0)*	1. 0 (0.0–1.0)	0.0 (0.0–1.5)
HADS Anxiety	6.5(5.0–11.0)	3.0 (1.2–3.0)*	6.6 ± 4.0	8.2 ± 4.8
HADS Depression	4.0 (3.0–7.8)	3. 0 (1.2–4.0)	8.1 ± 5.4	7.3 ± 4.1

^*^P < 0.05

*MRC* Medical Research Council, *CAT* COPD assessment test, *CCQ* Chronic COPD questionnaire, *HADS* Hospital anxiety and depression scale

**Table 4:** Exercise capacity at baseline and at the completion of the study

Parameter	Intervention group (n = 20)	Control Group (n = 20)		
	Baseline	Follow-Up	Baseline	Follow-ups
6-min walk test				
Distance (m)	409.8 ± 61.3	461.6 ± 50.0*	396.7 ± 94.2	386.6 ± 92.0
∆ SPO_2_ (%)	1.6 ± 3.4	0.9 ± 2.1	0.2 ± 1.8	1.2 ± 2.8
∆ HR (beats/min)	16.2 ± 10.9	14.1 ± 11.8	13.5 ± 9.9	10.1 ± 8.0
∆ SPB (mmHg)	17.0 ± 13.7	22.6 ± 22.2	16.8 ± 14.6	9.9 ± 7.6
∆ DBP(mmHg)	6.0 ± 13.8	7.8 ± 11.1	10.1 ± 9.4	5.8 ± 7.4
∆ Borg D (pts)	2.4 ± 2.2	0.6 ± 1.0*	2.3 ± 2.2	1.8 ± 2.1
∆ Borg E (pts)	4.6 ± 2.9	1.6 ± 1.5	4.8 ± 2.8	4.1 ± 3.2
Incremental shuttle walking test				
Distance (m)	223.0 ± 63.1	265.0 ± 70.5	216.0 ± 88.2	206.5 ± 76.7
∆ SPO_2_ (%)	− 2.5 ± 3.5	− 1.3 ± 2.3	− 0.8 ± 1.9	− 1.1 ± 1.6
∆ HR (beats/min)	10.2 ± 8.2	14.3 ± 12.3	12.2 ± 9.5	12.3 ± 13.1
∆ SPB (mmHg)	14.2 ± 16.3	15.2 ± 10.0	15.5 ± 9.9	3.9 ± 20.5
∆ DBP (mmHg)	5.3 ± 12.5	3.4 ± 8.4	4.8 ± 9.6	1.45 ± 5.1
∆ Borg D	1.8 ± 1.5	0.8 ± 1.2	1.6 ± 1.3	1.3 ± 1.2
∆ Borg E	4.6 ± 2.5	1.1 ± 1.4	3.6 ± 2.0	3.8 ± 2.5

∆-Baseline to end-of-exercise change

*SPO*_2_ peripheral oxygen saturation, *HR* heart rate, *SBP* systolic blood pressure, *DBP* diastolic blood pressure, *Borg D* borg dyspnoea scale, *Borg E* borg exertion scale

^*^P < 0.05

^a^Median (IQR)

## Discussion

This is the first prospective study conducted in Sri Lanka designed to explore the feasibility of devising a pulmonary rehabilitation program for patients with COPD in a low resource setting and to observe its effects. The study has three major findings. First, it is possible to establish a safe pulmonary rehabilitation program for patients with COPD using limited resources. Second, patients enrolled in the program experienced significant improvements in exercise capacity and decreases in symptom burden. Third, a likely obstacle which contributes to patient enrollment in pulmonary rehabilitation conducted in a LMIC such as Sri Lanka is the unawareness of the potential benefits of such program among patients and in the population at large.

### Feasibility of pulmonary rehabilitation in a low resource setting

Out of 204 patients screened, 136 (66.7%) patients were eligible for pulmonary rehabilitation yet, only 54 of them (39.7%) participated in the study, and only 40 (29.4%) eligible patients completed it. Reasons for lack of participation included no interest (25 patients, or 18.4% of eligible patients) and failure to attend baseline assessment due to discouragement by family members (42 patients, or 30.9% of eligible patients). Patients reported good compliance with unsupervised home exercises. Additionally, many patients in the intervention group experienced clinically meaningful improvements in respiratory symptoms, and exercise capacity.

The main purported reason for eligible patients to decline participation in the study was discouragement by family and friends. This observation underscores the need for educating both patients and the community at large about the purpose and the potential benefits of pulmonary rehabilitation in COPD. We explained to our study participants the potential benefits of pulmonary rehabilitation and told them that the cost of transport would be reimbursed. In the study, however, we did not take steps to educate family members or the local community on the importance of pulmonary rehabilitation in COPD.

Once our patients decided to attend the baseline visit, their intervention dropout rate was only 9.3%. This figure compares favourably with the reported 29% pulmonary rehabilitation dropout rate in the UK [26]. Whether such difference is the result of high patient satisfaction with our minimally-resourced pulmonary rehabilitation remains to be determined.

Nearly all patients eligible for pulmonary rehabilitation were males. This is not surprising considering that in Sri Lanka smoking is more prevalent among males than females [27]. Such observation, of course, does not mean that Sri Lanka women are not at risk of developing COPD, either due to smoking or occupational exposure to smoke, and, as such may also need pulmonary rehabilitation.

### Functional effects of pulmonary rehabilitation delivered in a low resource unit

The mean rehabilitation-associated improvements in CCQ and CAT scores in our intervention group were about 4 to fivefold the MCID [17, 18]. These results are superior to those reported in previous studies [16–18]. Our positive results cannot be attributed to longer duration of rehabilitation – in our study duration of pulmonary rehabilitation was shorter (6 weeks) than in previous studies. Beside success of pulmonary rehabilitation, several other mechanisms could have contributed to our positive results. These include investigators’ and patients’ bias due to lack of blinding or the fact that due to high illiteracy rate, the CCQ and CAT were administered by one investigator.

Both HADS-anxiety and HADS-depression scores were low at baseline. Therefore, it is not surprising that participants did not experience improvements in anxiety and depression (ceiling effect).

At the conclusion of the program, patients in the intervention group experienced significant improvements in walking distances and dyspnea on exertion. Forty-five percentage of these patients reached the MCID of 54 m [21] for the 6MWD. For the ISWD, 50% of patients in the rehabilitation group reached the MCID of 35 m [23]. The mean changes in ISWD and 6MWD of this group were comparable or better than the mean improvements reported in a 2015 Cochrane systematic review [28].

## Limitations

This study had several limitations. We did not conduct proper randomization of participants to study groups. Neither investigators nor patients were blinded to study interventions. The various questionnaires used were translated into Tamil, but have not been validated in this language. The study is a single-site investigation. Accordingly, our findings may not be generalizable across Sri Lanka. Compliance documentation of unsupervised home exercises was based on patients’ recall and not on objective monitoring.

## Conclusion

A simple and clinically beneficial pulmonary rehabilitation program for patients with COPD can be effectively and safely implemented in a low-resource setting. Our findings support the conduct of a future fully powered trial to determine pulmonary rehabilitation effectiveness and cost-effectiveness in Sri Lanka.

## Data Availability

The datasets used and/or analyzed during the current study are available from the corresponding author on reasonable request.

## Abbreviations

CAT: COPD assessment test
CCQ: Chronic COPD questionnaire
COPD: Chronic obstructive pulmonary disease
GOLD: Global initiative for chronic obstructive lung disease
HADS: Hospital anxiety depression scale
HRQoL: Health related quality of life
IQR: Inter quartile range
ISWD: Incremental shuttle walk distance
ISWT: Incremental shuttle walk test
LMIC: Low- and middle-income Countries
MCID: Minimal clinically important difference
MRC: Medical research council
SD: Standard deviation
6MWT: Six Minute walk test
6MWD: Six minute walk distance
